# Editorial of Special Issue “Cytoplasmic Delivery of Bioactives”

**DOI:** 10.1007/s11095-022-03290-2

**Published:** 2022-05-23

**Authors:** Zimei Wu

**Affiliations:** The University of Auckland, Auckland, New Zealand

**Keywords:** cytoplasmic delivery, endocytosis, endosome escape, exocytosis, nanostructure

Most of the therapeutic agents are designed to exert their pharmacological actions by modulating intracellular components of the target cells. However, many of these agents particularly macromolecules such as proteins and nucleus acids (DNA, RNA, and CRISPR/Cas9 systems) are unable to enter cells via passive diffusion. In other cases, small drug molecules may only reach the target cells at a suboptimal level due to their wide and nonspecific tissue distribution. In this context, nanotechnologies have gained major momentum as the most investigated approach to overcome the cellular barriers where a large payload can be delivered into the cells via various endocytic pathways. Endocytosis is a common phenomenon by which extracellular nanoparticles (and macromolecules) gain cell entry through membrane invaginations, leading to the formation of endosomes. However, a major bottleneck associated with this nanoparticle-mediated intracellular delivery approach is the sequestration of these particles in endosomes and later converged upon lysosomes where the payload may be degraded (1). In addition, the internalized nanoparticles trapped in the early endosome can be expelled (exocytosis) to the outside of the plasma membrane through the recycling route (2). As a result, only a small proportion of the drug molecules are released into the cytoplasm, and subsequently available to their action target at subcellular organelles: nucleus, mitochondria, and ribosomes. Therefore, cytoplasmic release through ‘endosome escape’ by regulating intracellular trafficking of the nanoparticles has been considered a key determinant of the efficacy of a therapeutic cargo (3, 4).

Recent advances in nanotechnology and nanoscience have enabled innovations in drug delivery. For example, numerous pH-sensitive or redox-sensitive nanocarriers by exploiting the intracellular microenvironments as endogenous stimuli (1, 5) have demonstrated their great potential to achieve cytoplasmic delivery with potentized pharmacological effects. This Special Issue themed on “Cytoplasmic delivery of bioactives” is dedicated to recognizing the recent progress in this field, bringing together researchers in drug delivery, bioengineering, and beyond to discuss advances and share their exciting findings alongside new insights into the topics.

## Highlights of the Special Issue

The Special Issue is composed of six reviews and six original research articles from several research groups. Some of the delivery strategies and applications are highlighted below.

### Review Articles

Wu and Li analyzed the lipid nanoparticle (LNP)-mediated cytoplasmic delivery strategies and elaborated the state-of-the-art nanotechnology that effectively breaks the cellular barriers and delivers COVID-19 messenger RNA (mRNA) vaccines to the target, ribosomes (6). Xu (7) offered a compelling analysis of the strategies to achieve cytoplasmic delivery of bioactives using inorganic nanoparticles. In their paper, various proposed mechanisms by which nanostructures undergo endosome escape were elaborated, including the salt osmotic effect and gas blast effect that explicitly belong to inorganic nanoparticles. Butt *et al*. (8) thoroughly reviewed the current understanding of the endosomal escape mechanisms of nanocarriers and offered their expert opinions on the design of polymeric micelles to achieve cytoplasmic delivery. Su and Zhang *et al*. (9) contributed a comprehensive update on the strategies to enhance cytoplasmic delivery of various types of bioactive molecules to immune cells and the cellular organelles. This included dendritic cells, macrophages, neutrophils, and T cells. Remarkably, immune cells have become attractive delivery platforms by taking advantage of their instinctive nature in surveillance of and fighting infection, inflammation, and cancer. On the other hand, intracellular infection with different pathogens (bacteria, virus and parasite) has posed a health risk worldwide, necessitating intracellular delivery of antimicrobials into the host/infected cells such as macrophages. Yeo and collaborators (10) conducted a timely meta-analysis of intracellular delivery strategies to target the pathogens in host cells. The review shed light on the trend over the past decades in intracellular delivery approaches using a wide range of micro-and nanostructures (liposomes, micelles, nanogels, polymeric nanoparticles, and dendrimers) to eradicate intracellular pathogens. Conversely, Pandey *et al*. (11) surveyed the recent advances in using bacteria-based systems for tumor-targeted drug delivery, including probiotic bacterial bodies (alive or ghost), and bacteria-derived ‘minicells’. These drug delivery vectors possess some unique advantages, for example, their natural immune-stimulating abilities and inherent tropism for the hypoxic tumoral cores.

### Original Research Articles

Nanoscale delivery systems enter cells via divers endocytic pathways, depending on particle size, shape, materials, surface charge, and modification. The use of a specific ligand for functional coating can dramatically increase the cellular uptake by targeted cells. Fucoidan, a polysaccharidic ligand for adhesion molecule P-selectin with additional anticancer properties, has gained attention for surface coating of nanoparticles for cancer drug delivery. In this collection, Vauthier’s team (12) studied the endocytic pathways of fucoidan-nanoparticles by macrophage (J774A) with aid of five endocytic inhibitors (chlorpromazine, genistein, cytochalasin D, methyl-ß-cyclodextrin, and nocodazole). The research revealed the significant impact of the polymer density and architectures at the surface of nanoparticles (‘mushroom’ *vs* ‘brush’) and their interplay with the particle size and surface charge on the endocytic pathways before reaching the cytosol. Bremmell and co-workers (13) investigated two cationic polymers, a branched polyethyleneimine (bPEI) and a fourth-generation polyamidoamine (PAMAM) dendrimer for their intracellular delivery efficiencies of small interference RNA (siRNA). The PAMAM dendrimer and PAMAM/siRNA complexes had a higher cell uptake transfection efficiency, and cytotoxicity compared with bPEI and bPEI/siRNA complexes, correlated with their interactions with an immobilized lipid membrane model. The two systems demonstrated different cytoplasmic distribution patterns. In the research work by Ma (14), dual-drug loaded pH-sensitive micelles were developed for effectively removing methicillin-resistant *Staphylococcus aureus* (MRSA) bacterial biofilm and meanwhile promoting wound healing. The small size (30 nm) biocompatible micelle system was able to penetrate biofilm and rapidly release the encapsulated antimicrobial (rifampicin) in response to the acidic microenvironment killing the bacteria. Meanwhile cytoplasmic delivery of Quercetin to epithelial cells via multiple endocytic pathways accelerated cell proliferation, which is crucial for wound healing. Wu and co-workers (15) reported calcium acetate enabled remote loading of a weakly acidic dinitrobenzamide mustard prodrug into pH-sensitive liposomes (PSL) (drug loading 30% or drug to lipid ratio almost 1:2; w/w). The cytotoxicity to a breast cancer cell line of PSL was 21- and 141-fold more potent than non-pH-sensitive liposomes and the free drug, respectively. For cytoplasmic delivery, the nanocarrier must carry an adequate payload to achieve the therapeutic effect. This study also demonstrated that increase the drug content in liposomes dramatically increased the drug cytotoxicity. In mice, a single treatment with PSL-SN25860 almost ablated all clonogenic tumor cells as observed in an ex vivo assay. Live cell imaging revealed the calcium ions inside PSL, but not in non-pH-sensitive liposomes, induced endo-lysosome rupture, presumably through the proton sponge effect, hence augmented cytosolic delivery. Lyophilization is a commonly used approach to enhance the shelf-life of protein drugs, representing a important step for their clinical application. Guanidinium-functionalized cationic poly(oxanorbornene)imide (PONI-Guan) developed by Rotello’s team is an effective vector for the direct cytosolic delivery of proteins (16). They investigated the lyophilized protein-PONI-Guan self-assembled nanocomposites and demonstrated direct cytosolic delivery of model proteins in several cell lines. The effective cytoplasmic delivery of the polymer-protein complex was also verified using an antitumor model protein in an *in vitro* cytotoxicity study. Finally, Zhang (17) drew their expertise in developing a novel fluorescent probe for specific imaging of the cytoplasmic organelle, lipid droplet (LD) in diverse cells. The specific LD-imaging nature was validated using the commercialized LD probe and other probes for organelles (nucleus, lysosome, mitochondria, and peroxisome) by imaging colocalization analysis. Interestingly, upon light irradiation the LD probe generated a lethal dose of reactive oxygen species in the cells, suggesting its potential in LD-targeted photodynamic therapy. Lipid droplets are considered as intracellular storage for lipids and are recently recognized as vital hubs of cellular metabolism via interactions with other organelles such as mitochondria (18).

## Cytoplasmic Delivery and Beyond

Cytoplasmic delivery is a comprehensive topic, involving a full understanding of interactions of the nanostructures with the cells including 1) endocytosis, 2) intracellular trafficking, and 3) exocytosis. To date we have gained great understanding of the factors that promote endocytosis of nanomedicine or macromolecules (19–21). By this Special Issue collection, despite just being the *tip* of the *iceberg,* we aim to gain insights into the design of biomaterials and nanostructures, their intracellular trafficking mechanisms as well as some commonly used research methodologies.

It is worth noting that cell-penetrating peptides and some nanostructures such as reversed micelles are internationalized via non-endocytic uptake pathways via pore-forming or fusion with the cell membrane, directly transporting their cargos into the cytoplasm (22). In addition to the delivery approaches discussed in this Special Issue, dequalinium-based liposome-like vesicles (DQAsomes) are a special type of nanostructures that specifically deliver bioacitve molecules to mitochondria (23).

Transcytosis (polarized exocytosis) has been recently recognized as a new paradigm for nanoparticles transported through the endothelial layer in the tumor blood vessel to the tumors (*versus* through the inter-endothelial gaps via the Enhanced Permeability and Retention effect) (24). On the other hand, as mentioned early, exocytosis of nanoparticles by the targeted cells can be a major roadblock for gene therapy where cytoplasmic delivery is desirable (2). It has been reported that internalized mRNA-loaded lipid nanoparticles were re-packaged into extracellular vesicles (exosomes) and secreted from the recipient cells (25). Recent research found that the pH-sensitivity of liposomes increased cytoplasmic delivery and suppressed the degree of exocytosis of liposomes, as well as the transcytosis through an *in vitro* brain vascular endothelial monolayer model (26). Therefore, this Special Issue may also help open up new discussion avenues around these important topics.

Another important note is that while cytoplasmic delivery is considerable for many types of biological agents with action target in cytosol or organelles, the choice of a delivery strategy depends on the purpose and mode of action. For example, ‘endosome entrapment’ may be a preferential pathway for some therapeutic agents, e.g. antibody-drug conjugate and some prodrugs that are designed to be activated by the lysosomal hydrolytic enzymes (27). Lysosome-targeting via the endocytosis pathway provides a unique opportunity for the effective treatment of lysosome-related diseases especially lysosomal storage disorders, and neurodegenerative conditions (28).

## Final Remarks

Recent advancements in biotechnology have yielded an increasing number of therapeutic candidates including macromolecules, bringing new hopes to patients with more effective treatment options. A particular example is the recent success of mRNA vaccines which helped to save numerous lives from COVID-19. This ground-breaking development has opened a new chapter to tackle many unmet medical needs using nucleic acid drugs, and protein replacement therapies that necessitates the use of cytoplasmic delivery.

We expect that this Special Issue will enhance our understanding of the factors that govern the intracellular trafficking of nanomedicine and macromolecules. In addition, the research may shed some light on how to consolidate anticancer nanomedicines following the initial hype thus increasing their clinical translation. There are many physical and biological barriers required to be circumvented to reach the target cells, necessitating multidisciplinary collaborative effect to keep improving drug delivery science and technologies. Let us be encouraged by the vision and optimisms of Professor Kinam Park, a giant in drug delivery research – ‘the future is bright’ (29).

Finally, I would like to take this opportunity to personally thank all authors, peer reviewers, and the journal’s editors for their invaluable participation.

Zimei Wu

Guest editor of Special Issue Cytoplasmic Delivery of Bioactives

School of Pharmacy, The University of Auckland, New Zealand
